# Suicide versus Accidental Death by Autoerotic Asphyxiation in a Patient Receiving Intravenous Ketamine for Depression

**DOI:** 10.1155/2022/1104668

**Published:** 2022-04-26

**Authors:** Jeremy Weleff, Kelly Bryant, Alexsandra Kovacevich, Brian S. Barnett

**Affiliations:** ^1^Department of Psychiatry and Psychology, Center for Behavioral Health, Neurological Institute, Cleveland Clinic, Cleveland, OH, USA; ^2^Cleveland Clinic Lerner College of Medicine at Case Western Reserve University, EC-10 Cleveland Clinic, 9501 Euclid Ave., Cleveland, OH, USA

## Abstract

**Background:**

Clinical trials have demonstrated that subanesthetic intravenous ketamine exerts antidepressant effects lasting a week or longer postinfusion, as well as antisuicidal effects starting approximately 4 hours postinfusion and lasting 72 hours or longer. These findings have generated considerable enthusiasm within psychiatry. However, reports of treatment-emergent suicide attempts and completed suicides in some patients receiving ketamine or the ketamine enantiomer esketamine have begun to emerge. Here, we contribute to the small literature on suicide-related adverse events and ketamine with an unusual case of a patient who died either by suicide or accidental death via autoerotic asphyxiation approximately four days after a ketamine infusion. *Case Presentation*. The patient was a 28-year-old man with major depressive disorder, generalized anxiety disorder, panic disorder, obsessive compulsive disorder, autism spectrum disorder without intellectual disability, attention deficit hyperactivity disorder, hypothyroidism, low testosterone, and sleep apnea referred for management of treatment resistant depression. His depression briefly remitted with ketamine, and suicidality briefly disappeared. However, these improvements were short-lived. Four days after his seventh and final scheduled ketamine infusion, the patient was found dead, presumably due to autoerotic asphyxiation. Interestingly, ketamine use has been reported in association with autoerotic asphyxiation. However, given our patient's recent severe suicidality, methods of his past suicide attempts, and family history of suicide, death from suicide seems more likely. *Discussion*. Here we consider the possibility of whether ketamine may have contributed to the patient's possible suicide, either via a direct worsening of his suicidality or psychological withdrawal following cessation of treatment, given recent concerns about psychological withdrawal's potential role insuicides following esketamine treatment.

**Conclusions:**

Though we are uncertain about the patient's cause of death, this case provides an opportunity to highlight important gaps in our understanding of the suicide-related risks of subanesthetic intravenous ketamine treatment for mood disorders and suicidality.

## 1. Introduction

Repeated clinical trials have demonstrated that treatment with subanesthetic intravenous ketamine, usually infused at 0.5 mg/kg over 40 minutes, is associated with rapid improvement in depression that can endure one week or longer [1]. Clinical trials have also demonstrated reduced suicidal ideation in patients treated with subanesthetic intravenous ketamine beginning four hours postinfusion and lasting for up to 72 hours postinfusion [2, 3], with one study demonstrating reduced suicidality at 6 weeks posttreatment [4]. Intriguingly, the antisuicidal effects of ketamine appear to be at least partially independent of its antidepressant effects [5].

With limited options available to psychiatrists for effectively addressing suicidal ideation quickly, the discovery of ketamine's rapid antisuicidal properties [6], as well as a potential new approach for treating depression and suicidality via modulation of glutamatergic transmission [7–9], has generated considerable enthusiasm within psychiatry. However, many questions remain to be answered regarding ketamine's antisuicidal property, including to what degree this effect extends to intramuscular and oral routes of administration [10] and whether the severity of a patient's suicidality or their risk for suicide may worsen once ketamine's effects have worn off.

Another important consideration in the use of ketamine in patients with suicidality is the fact that treatment emergent suicide attempts and suicides have been reported in some patients receiving ketamine and the ketamine enantiomer esketamine [11–13]. Given the rarity of these events and the limited nature of research into ketamine's antidepressant and antisuicidal effects, it is still unknown whether ketamine could actually be causing or worsening suicidality in some vulnerable individuals and, if so, who might be at risk for these adverse events. While seemingly contradictory, an antidepressant increasing suicidality is not a new phenomenon, having been observed rarely in some patients, particularly children and adolescents, taking selective serotonin reuptake inhibitors [14]. With these considerations in mind, here, we report an unusual case involving the death of a patient by either suicide or autoerotic asphyxiation approximately four days after his seventh subanesthetic ketamine infusion for treatment resistant depression.

## 2. Case Report

Mr. G. was a 28-year-old man with major depressive disorder, generalized anxiety disorder, panic disorder, obsessive compulsive disorder, autism spectrum disorder without intellectual disability, attention deficit hyperactivity disorder, hypothyroidism, low testosterone, and sleep apnea referred for management of treatment resistant depression. He had five past suicide attempts, including one by medication overdose, one by inhalation of bleach and ammonia, one by self-suffocation with a plastic bag, and one, a month before referral, by wrapping tie-down straps around his neck in an effort to suffocate himself. Family history was notable for depression in his mother and paternal aunt and suicide in his maternal cousin. Previous medications included alprazolam, aripiprazole, buspirone, citalopram, clomipramine, divalproex sodium, duloxetine, escitalopram, fluoxetine, fluvoxamine, lamotrigine, methylphenidate, mirtazapine, modafinil, olanzapine, paroxetine, selegiline (transdermal), topiramate, trazodone, venlafaxine, vortioxetine, and zolpidem. With the exception of divalproex sodium, which was discontinued due to leg swelling, the patient's other medications were discontinued due to lack of efficacy for his various psychiatric conditions. Of note, the patient had never received a trial of lithium for his suicidality.

At time of referral, Mr. G. was taking armodafinil 250 mg daily, clonazepam 1 mg daily as needed for anxiety, dexmethylphenidate extended release 10 mg daily, quetiapine 100 mg nightly, testosterone 20.25 mg/1.25 gr (1.62%) transdermal gel pump (3 pumps per day), metformin 500 mg daily, and levothyroxine 75 mcg daily. Of note, he was not on an oral antidepressant, which is recommended for patients receiving treatment with the ketamine enantiomer esketamine in that medication's package insert (Janssen Pharmaceuticals [15]).

At initial evaluation, Mr. G. reported sadness, anhedonia, anergia, anorexia, and insomnia. He reported no past mania, psychosis, or substance misuse. Montreal Cognitive Assessment (MoCA) score was 28, and Montgomery-Åsberg Depression Rating Scale [16] (MADRS) score was 43 on initial evaluation (score > 34 is considered severe depression). Of note, the patient reported suicidal ideation with plan, but no intent, to suffocate himself via carbon monoxide, scoring 5/6 on the MADRS suicidality item.

Approximately one week postevaluation and seven weeks before Mr. G's death, electroconvulsive therapy (ECT) was initiated. Just prior to his first ECT session, the patient was administered the Columbia Suicide Severity Rating Scale, on which he scored an 18/25 for most severe lifetime suicidal severity and 16/25 for most severe past month suicidal severity, with both scores being in the “severe” range. After nine ECT treatments, his MADRS score dropped to 25 (≥50% reduction in MADRS with initial score of >10 is considered treatment response), but depression and thoughts of suicide via multiple methods, which he did not feel like sharing with the team, without intent persisted.

Due to his limited treatment response, Mr. G. subsequently underwent an acute series of seven intravenous ketamine infusions over three weeks. He initially appeared to have a robust response, with his MADRS score decreasing from 33 preinfusion one to 21 preinfusion two and his MADRS suicidality item score falling from four (“Probably better off dead. Suicidal thoughts are common, and suicide is considered as a possible solution, but without specific plans or intention.”) to two (“Weary of life. Only fleeting suicidal thoughts.”). After infusion three, Mr. G. reported cessation of suicidal thoughts for the first time in his adult life to his therapist. His MADRS score reached a nadir of 10 (indicating remission) one day after infusion four, and his score on the MADRS suicidality question dropped to zero (“Enjoys life or takes it as it comes.”). However, four days later, just prior to infusion five, his MADRS score rose to 14, and the MADRS suicidality item score rose back to two. Just before his seventh and final scheduled ketamine infusion, his MADRS score was 16, and Mr. G. again scored two on the suicidality item. Four days later, Mr. G. was found dead with a belt around his neck. His body was discovered in the morning, and he had last been seen the previous evening, putting time of death somewhere between three-and-a-half and four days postinfusion.

Mr. G.'s official cause of death was ruled accidental death by autoerotic asphyxiation, a masochistic sexual behavior that involves achieving sexuaul arousal by restricting the brain's access to oxygen, most often via ligature around the neck [17]. The patient's family was unaware of any such past behavior. Notably, approximately one week before ketamine initiation, Mr. G. had reported being distressed by loss of sexual desire and trouble achieving orgasm via a standard screening instrument.

Because of Mr. G.'s recent severe suicidal ideation, the manners in which he had previously attempted suicide, and his family history of suicide, our team believes his cause of death was more likely due to suicide, rather than accidental death. Assuming his death was a suicide, application of the Naranjo Adverse Drug Reaction Probability Scale [18], revealed a “possible” probability of ketamine contributing to his death (total score of 3 out of 13 (+1 for previous conclusive reports on this reaction, +2 for adverse event appearing after suspected drug was administered, -1 for potential alternative causes of the adverse event, and +1 for the adverse event being confirmed by objective evidence).

## 3. Discussion

It is unclear to us whether Mr. G. completed suicide or died accidentally from autoerotic asphyxiation. Regarding the latter possibility, ketamine intoxication, perhaps due to enhancing sexual excitement/orgasmic experience or producing behavioral disinhibition, has been reported in autoerotic asphyxiation and associated accidental deaths [19, 20]. Given ketamine's half-life of 2-4 hours [21], it is possible that Mr. G. could have discovered novel autoerotic asphyxiation behavior soon after a ketamine infusion and continued to engage in this behavior on noninfusion days, possibly as a means of dealing with his difficulties achieving orgasm. Though intriguing, we think the possibility of ketamine producing or reinforcing autoerotic asphyxiation behavior that may have resulted in accidental death is unlikely. A more probable explanation is that Mr. G. was engaging in this behavior prior to starting ketamine treatments, and his accidental death from it at the conclusion of his acute ketamine infusion series was simply a coincidence. However, given Mr. G.'s history of serious suicidality, family history of suicide, and the similar methods employed in his previous suicide attempts, including wrapping tie-down straps around his neck a month prior to referral, we think it more likely that the official cause of death was incorrect and that he died from suicide. If so, his suicide would appear to have occurred just outside the typical timeframe for ketamine's antisuicidal efficacy [3].

Though Mr. G.'s possible suicide may have simply been an unfortunate inevitability that occurred just as the antisucidial effects of ketamine wore off, it is also possible that ketamine may have played a role. Suicide attempts and completed suicides in patients who have responded well to ketamine have been previously reported, including the case of a patient who attempted suicide one week after discontinuing intranasal ketamine [11]. Interestingly, that patient's depression remitted after restarting ketamine. Additionally, treatment emergent suicide attempts and completed suicides have been reported in patients receiving ketamine and esketamine, with a 2021 systematic review of the effects of esketamine and intravenous ketamine on patients with baseline suicidality in randomized clinical trials revealing that 2.37% of participants receiving either intravenous ketamine or esketamine had a treatment emergent suicide attempt during the double-blind phase of the trials compared to 1.48% receiving placebo, though there were no completed suicides in either group [13]. In the follow-up phase of those clinical trials, 3.11% of participants receiving either IV ketamine or esketamine attempted suicide compared to 1.43% of placebo patients, though completed suicides were higher in the placebo group (0.31% for esketamine/ketamine vs. 1.43% for placebo group). A recent study of postmarketing safety data on esketamine that analyzed reports made to the United States Food and Drug Administration Adverse Event Reporting System found a 24.0 increased odds for development of suicidal ideation during treatment with esketamine and a 5.75 increased odds for completed suicide attempt compared to other medications [12].

If Mr. G.'s death was a suicide, another important consideration is whether ketamine withdrawal may have played a role. In 2019, prominent American psychiatric researcher Alan Schatzberg raised questions about the potential role of a protracted psychological withdrawal reaction in the suicides of patients recently treated with esketamine, pointing out that in one randomized clinical trial of intranasal esketamine there were three suicides 4-20 days following last esketamine dose and none in the placebo group [22]. Notably, two of the participants who committed suicide had not previously expressed suicidality during the trial. One further consideration, given increasing positive media coverage of ketamine as a novel treatment for depression and suicidal ideation [23], is that a “disappointment reaction”, a phenomenon where patients are left despondent after being failed by a novel treatment that they placed their hopes in [24], may have worsened Mr. G.'s suicidal ideation.

## 4. Conclusions

Though we are uncertain about Mr. G.'s cause of death, this case provides an opportunity to highlight important gaps in our understanding of the suicide-related risks of subanesthetic intravenous ketamine treatment for mood disorders and suicidality. Mr. G.'s possible suicide while undergoing treatment with subanesthetic intravenous ketamine and reports of treatment emergent suicide attempts and completed suicides occurring in other patients receiving ketamine for mood disorders and suicidality suggest that discussing the possibility of new onset or worsening suicidal ideation during ketamine treatments may be a necessary component of the informed consent process for treatment with ketamine. Additionally, while some clinics offer ketamine for depression as frequently as once a week for maintenance treatment, given the short acting nature of ketamine's antisuicidal effects, this cadence still leaves time for suicidality to recur in between infusions for patients with baseline suicidality. Therefore, clinicians should not only caution patients about the possibility of a rare worsening of suicidality while undergoing ketamine treatments but also routinely ask patients whether their suicidality has worsened or changed in quality upon its likely return in between treatments. Clinicians should also encourage patients to reach out during periods in between infusions if their suicidality worsens to discuss possible interventions and whether continuing ketamine is the best treatment option.
